# Deletion of 12/15-Lipoxygenase Alters Macrophage and Islet Function in NOD-*Alox15^null^* Mice, Leading to Protection against Type 1 Diabetes Development

**DOI:** 10.1371/journal.pone.0056763

**Published:** 2013-02-21

**Authors:** Shamina M. Green-Mitchell, Sarah A. Tersey, Banumathi K. Cole, Kaiwen Ma, Norine S. Kuhn, Tina Duong Cunningham, Nelly A. Maybee, Swarup K. Chakrabarti, Marcia McDuffie, David A. Taylor-Fishwick, Raghavendra G. Mirmira, Jerry L. Nadler, Margaret A. Morris

**Affiliations:** 1 Department of Internal Medicine, Eastern Virginia Medical School, Norfolk, Virginia, United States of America; 2 Department of Pediatrics and Herman B Wells Center for Pediatric Research, Indiana University School of Medicine, Indianapolis, Indiana, United States of America; 3 Graduate Program in Public Health, Eastern Virginia Medical School, Norfolk, Virginia, United States of America; 4 Department of Microbiology, University of Virginia, Charlottesville, Virginia, United States of America; 5 Department of Medicine, University of Virginia, Charlottesville, Virginia, United States of America; 6 Department of Microbiology and Molecular Cell Biology, Eastern Virginia Medical School, Norfolk, Virginia, United States of America; University of Bremen, Germany

## Abstract

**Aims:**

Type 1 diabetes (T1D) is characterized by autoimmune depletion of insulin-producing pancreatic beta cells. We showed previously that deletion of the 12/15-lipoxygenase enzyme (12/15-LO, *Alox15* gene) in NOD mice leads to nearly 100 percent protection from T1D. In this study, we test the hypothesis that cytokines involved in the IL-12/12/15-LO axis affect both macrophage and islet function, which contributes to the development of T1D.

**Methods:**

12/15-LO expression was clarified in immune cells by qRT-PCR, and timing of expression was tested in islets using qRT-PCR and Western blotting. Expression of key proinflammatory cytokines and pancreatic transcription factors was studied in NOD and NOD-*Alox15^null^* macrophages and islets using qRT-PCR. The two mouse strains were also assessed for the ability of splenocytes to transfer diabetes in an adoptive transfer model, and beta cell mass.

**Results:**

12/15-LO is expressed in macrophages, but not B and T cells of NOD mice. In macrophages, 12/15-LO deletion leads to decreased proinflammatory cytokine mRNA and protein levels. Furthermore, splenocytes from NOD-*Alox15^null^* mice are unable to transfer diabetes in an adoptive transfer model. In islets, expression of 12/15-LO in NOD mice peaks at a crucial time during insulitis development. The absence of 12/15-LO results in maintenance of islet health with respect to measurements of islet-specific transcription factors, markers of islet health, proinflammatory cytokines, and beta cell mass.

**Conclusions:**

These results suggest that 12/15-LO affects islet and macrophage function, causing inflammation, and leading to autoimmunity and reduced beta cell mass.

## Introduction

Type 1 diabetes (T1D) is a complex autoimmune disease in which immune cells react against insulin-producing beta cells within the pancreatic islets of Langerhans [1], [2], leaving affected individuals dependent upon exogenous insulin for life, and at high risk for developing serious cardiovascular and microvascular complications. Female NOD mice develop spontaneous type 1-like diabetes that mimics human disease [3]. We previously showed that the *Alox15* gene locus contributes to diabetes pathogenesis in the NOD model using a congenic strain lacking *Alox15* (NOD-*Alox15^null^*) [4]. *Alox15* deletion leads to 98 percent protection from diabetes in female NOD mice. Furthermore, macrophage infiltration and CD4^+^ T cell infiltrates into the pancreas were significantly reduced in NOD-*Alox15^null^* mice [4].

The murine *Alox15* gene encodes the 12/15-lipoxygenase (12/15-LO) enzyme, which is involved in the oxygenation of arachidonic and linoleic acids to the inflammatory mediators 12-S-hydroperoxyeicosatetraenoic acid and 13-(S)-hydroxy-9Z11E-octadecadienoic acid. Unstable 12-S-hydroperoxyeicosatetraenoic acid is highly toxic, and is almost immediately converted to the more stable 12-HETE (hydroxyeicosatetraenoic acid) by glutathione peroxidase [5]–[7]. 12-HETE can activate a signaling cascade that can lead to cytokine-induced cell damage [8]. Downstream generation of IL-12 by 12/15-LO products presumably leads to T cell activation and phosphorylation of signal transducers and activators of transcription 4 (STAT4) [9], [10]. Phosphorylated STAT4 leads to the production and activation of many downstream proinflammatory cytokines, which can contribute to the destruction of pancreatic beta cells and the autoimmunity seen in T1D [8], [11].

Macrophages are a primary source of IL-12, and global deletion of 12/15LO in C57BL/6J mice has been demonstrated to reduce IL-12 production [12], [13]. Downstream effectors of the 12/15-LO pathway can lead to a positive feedback loop, perpetuating the inflammatory response [8]. Therefore, reduced activation of the IL-12/STAT4 cascade may be a potential mechanism for reduced autoimmunity and inflammation in NOD- *Alox15^null^* mice.

12/15-LO expression has also been seen in pancreatic beta cells of both humans and rodents [8], [14], [15]. It is possible that islet production of 12-HETE could lead to significant islet damage in the context of inflammatory stress, leading to beta cell dysfunction or loss of viability [14], [15]. Furthermore, recent evidence shows that NOD mice experience intrinsic beta cell dysfunction prior to diabetes onset [16]. When *Alox15* is specifically deleted in islets of mixed background mice, these mice are protected from developing type 1-like diabetes following streptozotocin treatment [17].

In this study, we performed experiments to further clarify the *in vivo* relevance and possible mechanisms for the role of 12/15-LO in T1D induction in NOD mice. We investigated the role 12/15-LO expression plays in the development of autoimmune responses in T1D, with a specific emphasis on macrophage expression. We also studied the effects of 12/15-LO expression on islet health and function. The results of our studies indicate a role for 12/15-LO in both macrophage and islet function in the development of T1D.

## Methods

### Ethics Statement

All mice were treated in accordance with the “Principles of laboratory animal care” (NIH publication no. 85–23), AAALAC, and IACUC guidelines at the Eastern Virginia Medical Center.

#### Mice

Female NOD/ShiLtJ (NOD, used at 4–16 weeks old) and NOD.CB17-*Prkdc^scid^*/J (NOD.*scid,* ordered at 8 weeks old from The Jackson Laboratory, Bar Harbor, ME), NOD-*Alox15^null^* (used at 4–16 weeks old), and NOD-*Alox15^null^.scid* mice (colonies maintained on-site, used at 8–10 weeks old) were housed under SPF conditions. *Scid* strains were fed antibiotic chow (TD.07194-Uniprim diet) every other week to minimize infections with environmental bacteria. Blood glucose of NOD mice was monitored twice weekly by a One Touch Ultra blood glucose monitor (Lifescan, Inc., Milpitas, CA) beginning at 10 weeks of age, and two consecutive daily readings above 250 mg/dL indicated overt diabetes.

### Islet & Acinar Isolation

Pancreatic islets from NOD and NOD-*Alox15^null^* mice were isolated by collagenase digestion and Histopaque (Sigma-Aldrich, St Louis, MO, USA) centrifugation using a modified version of a previously published protocol [18]. Islets were used the same day for qRT-PCR and Western blots.

### Cell Isolations

T and B cells were isolated from spleen by mouse subset positive selection kits from Stem Cell Technologies (Vancouver, BC, Canada). Resulting populations were >97% pure.

#### qRT-PCR

RNA and cDNA were prepared as described [19]. For quantitative measurement of most RT-PCR products, TaqMan probes (Applied Biosciences, Carlsbad, CA, USA) were used with Jump Start Taq Polymerase (Sigma-Aldrich). The PCR conditions for all genes except 12/15-LO were: denaturation at 95°C for 30 s, annealing and extension at 60°C for 60 s at 40 cycles. Reactions were performed in triplicate and data were normalized to the actin housekeeping gene. Expression levels were presented as either fold change of transcripts compared to time matched controls, or as 1/ΔCt. 12/15-LO mRNA expression was quantitatively measured as previously described [19] using SYBR green dye (Invitrogen, Carlsbad, CA, USA) with the following primer sequences: Forward, 5′-CTCTCAAGGCCTGTTCAGGA-3′; reverse, 5′-GTCCATTGTCCCCAGAACCT-3′ decreasing the annealing temperature to 60°C. Expression was normalized to β actin expression, and expressed as ΔCt.

### TH17 PCR Array

A mouse TH17 Array was purchased from SABioscienes (Frederick, MD, USA) and used as directed. Reactions were analyzed using a two-step cycling program on a Bio-Rad CFX96 real-time system (Hercules, CA, USA). Results were validated by quantitative RT-PCR, described above.

### Western Blotting

Islet protein extracts from 200 mouse islets (NOD and NOD-*Alox15^null^*) were isolated for western blotting (10% gel). 15 µg protein was used to ensure equal loading per lane, followed by blotting with rabbit actin (I-19)-R (Santa Cruz, Santa Cruz Biotechnology, CA, USA), and rabbit leukocyte 12-LO Ab [8].

### Thioglycollate-Induced Peritoneal Macrophages

16-week old female NOD and NOD-*Alox15^null^* mice were injected i.p. with 2 ml of 4% thioglycollate (Sigma-Aldrich). Cells were harvested three days later, allowed to settle for 3 hours, and cultured in RPMI with 10% FBS for 24 hours, followed by culture in RPMI with 0.2% BSA. After 4 days, cells were harvested and mRNA isolated for analysis by RT-PCR (described above). For protein assays, 12 week old NOD or NOD-*Alox15^null^* mice were used. Cells were allowed to adhere for 3 hours, then treated with LPS (10 ng/ml) and IFN-γ (100 ng/ml). Cells were harvested after 24 hours. Both supernatants and cell pellets were saved for analysis by ELISA.

### ELISA

IL-1β protein levels were measured in cell supernatants per the manufacturer’s instructions (eBioscience Platinum ELISA, San Diego, CA, USA). Total STAT4 protein was measured in cell lysates per the manufacturer’s instructions (My BioSource, San Diego, CA, USA). Data are presented as a ratio of pg of the specific protein tested compared to µg of total protein.

### Intracellular Flow Cytometry

IL-12p35 and IL-12p40 levels were measured in bone marrow macrophages. Cells were plated in 6 well plates, and incubated overnight. Subsequently, cells were either treated with IFN-γ (100 ng/ml) for 6 hours at 37°C, then LPS (10 ng/ml) overnight, or left untreated in media alone. Brefeldin A was added to both groups to prevent secretion of cytokines. Data are expressed as fold-change of stimulated cell expression over untreated cells, after accounting for staining in isotype-matched control antibodies.

### Immunofluorescent Staining of Frozen Tissues

Tissues were snap frozen for sectioning, and staining as described [4]. Stained slides were viewed on a Zeiss Axio Observer Z1 microscope, and analyzed with Zeiss AxioVision software (Carl Zeiss MicroImaging, Thornwood, NY, USA).

### Scoring of Insulitis

Briefly, frozen sections were stained as described above, and blindly scored for insulitis (level of mononuclear cell infiltration), granulation (level of insulin staining), and the presence of either CD4^+^ or Foxp3^+^ T cells as described previously [4]. The scoring scale ranges from 0 (no insulitis/granulation/T cells) to 3 (maximal insulitis/granulation/T cells).

### Beta Cell Area

Beta cell area was calculated as described previously [20], but with some modifications. Pancreata from 3–5 mice per group were rapidly dissected, fixed in 4% paraformaldehyde, paraffin-embedded, and longitudinally sectioned. Three sections per pancreas (approximately 75 µm apart) were immunostained for insulin and counterstained with hematoxylin as described previously [21]. Digital images were acquired on an Axio-Observer Z1 microscope fitted with an AxioCam high-resolution color camera. Relative beta cell area was calculated using Axio-Vision Software.

### Measurement of Total Serum Cytokines

Serum was collected following cardiac puncture at the time of euthanasia as described [22]. The Luminex system (Millipore) was used to analyze serum samples using a multiplex kit for detecting mouse cytokines and chemokines from Millipore (Billerica, MA, USA).

### Adoptive Transfer

Total splenocytes (20×10^6^ cells) from age-matched donors (13 weeks of age at donation) were injected via the lateral tail vein into either NOD.*scid* or NOD-*Alox15^null^*.*scid* mice, based on previous studies [23], [24]. Blood glucose was monitored pre- and post-transfer for up to 10 weeks to ascertain disease progression.

### Cytokine Production in Beta Cells

βTC3 cultured to near-confluency were either left untreated, or treated for 6 hours at 37°C with a triple cytokine cocktail containing IL-1β (0.5 ng/ml), TNF-α (1 ng/ml) and IFN-γ (10 ng/ml). Cells were harvested, mRNA was extracted, cDNA made, and the gene expression measured using RT-PCR as described above.

### Glucose Stimulated Insulin Secretion (GSIS)

MIN6 beta cells were used as previously described [25], but treated with 2.5 ng/ml of IL-12p70 as the experimental condition. Insulin concentrations were determined using an Insulin ELISA kit (MERCODIA AB, Uppsala, Sweden). The data is shown as insulin secretion normalized to total protein. To graph in this way we first used the Lowry protein assay to calculate the “Total protein” in µg/µl. We then converted the “Total insulin” secretion values from the ELISA to µg/µl based on a formula given in the manufacturer’s instructions. To get “Insulin secretion normalized to total protein”, we divided the value for “Total protein” by the value for “Total insulin” from corresponding wells.

### Statistics

The time to diabetes development was analyzed using the Kaplan-Meier method to construct survival curves. Breslow-Wilcoxon tests were used to determine whether differences between the 2 experiments/strains were significant. Statistical tests were performed using SAS 9.3 (the SAS Institute) with a significant level of alpha = 0.05. Additionally, statistically significant differences were determined by the use of two-tailed Student’s T test, where appropriate, or ANOVA followed by post-hoc testing. Significant differences in all cases were determined by p<0.05.

## Results

### Characterization of 12/15-LO in Immune Cells and Effects on Gene Expression

In order to characterize the mechanism of protection from diabetes progression in NOD- *Alox15^ null^* mice, we first investigated several aspects of the timing and cellular location of the *Alox15* gene expression in NOD mice, and how alterations in expression might affect inflammation. We concentrated on expression in two main cell types: macrophages and pancreatic islets.

**Figure 1:**
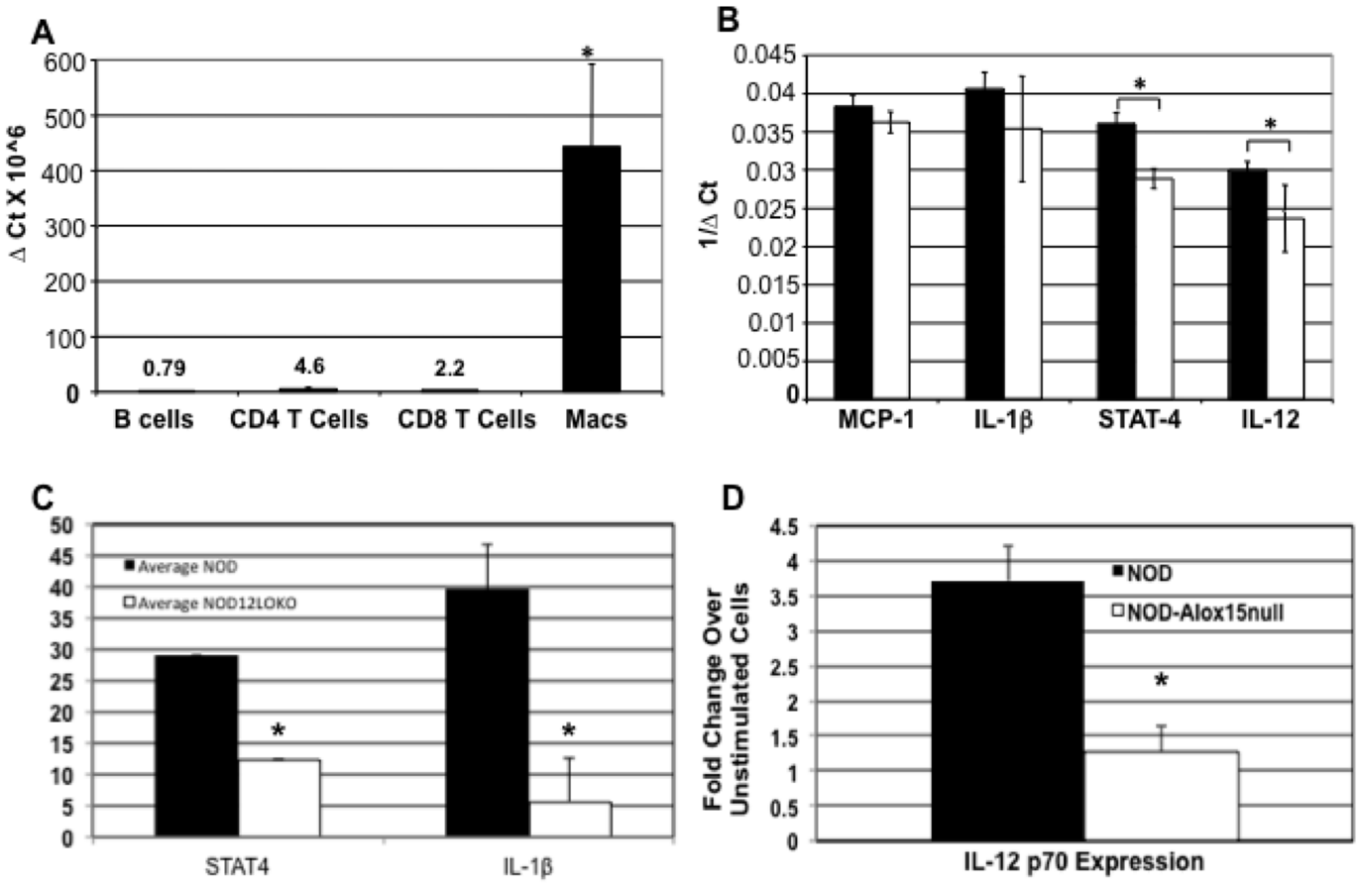
Expression of Macrophage 12/15-LO and downstream mediators. A) 12/15-LO expression of NOD mice macrophages versus lymphocytes. Data are shown as ΔCt X10^6^. B) Macrophage mRNA expression of downstream effectors of the 12/15-LO pathway. Thioglycollate-induced peritoneal macrophages of NOD (n = 5) and NOD-*Alox15^null^* (n = 3) mice were tested for mRNA expression of proinflammatory cytokines and STAT4 by qRT-PCR using Taqman probes. Data are shown as 1/ΔCt. Data represent 2 experiments with n = 4 mice per group. C) Protein levels for total STAT4 and IL-1β in thioglycollate-induced macrophages were measured in duplicate by ELISA (NOD, n = 5; NOD-*Alox15^null^*, n = 4). D) IL-12p70 levels in bone marrow macrophages were determined by intracellular flow cytometry (n = 3 for each strain. *p<0.05. Filled bars represent NOD samples. Filled bars represent NOD mice and open bars represent NOD-*Alox15^null^* mice.

Previously, we showed that macrophages of NOD mice clearly express 12/15-LO mRNA and protein, while 12/15LO expression in NOD-*Alox15^null^* macrophages was absent [4]. Here, we tested 12/15-LO mRNA expression levels in age-matched, isolated peritoneal macrophages, CD4^+^ and CD8^+^ T cells, and B cells. 12/15-LO was seen almost exclusively in macrophages compared to essentially undetectable levels in lymphocytes (Figure 1A). Therefore, macrophages, but not B and T cells, are most likely to participate in the generation of the *Alox15* gene products. We then investigated how 12/15-LO expression affected macrophage proinflammatory cytokines downstream of 12/15-LO, including MCP-1, IL-1β, STAT-4, and IL-12p40. STAT-4 and IL-12p40 mRNAs were significantly decreased in 12/15-LO deficient macrophages (Figure 1B). To confirm that similar results were seen in protein levels of the downstream mediators, we performed ELISAs (MCP-1, IL-1β, and STAT4) or intracellular staining (IL-12p70). NOD-*Alox15^null^* macrophages exhibited significantly lower levels of IL-1β, total STAT4 (Figure 1C), and IL-12p70 proteins (Figure 1D). MCP-1, similar to the mRNA levels, was not decreased in the NOD-*Alox15^null^* macrophages (data not shown).

### T Cell Function in the Absence of 12/15-LO

**Figure 2:**
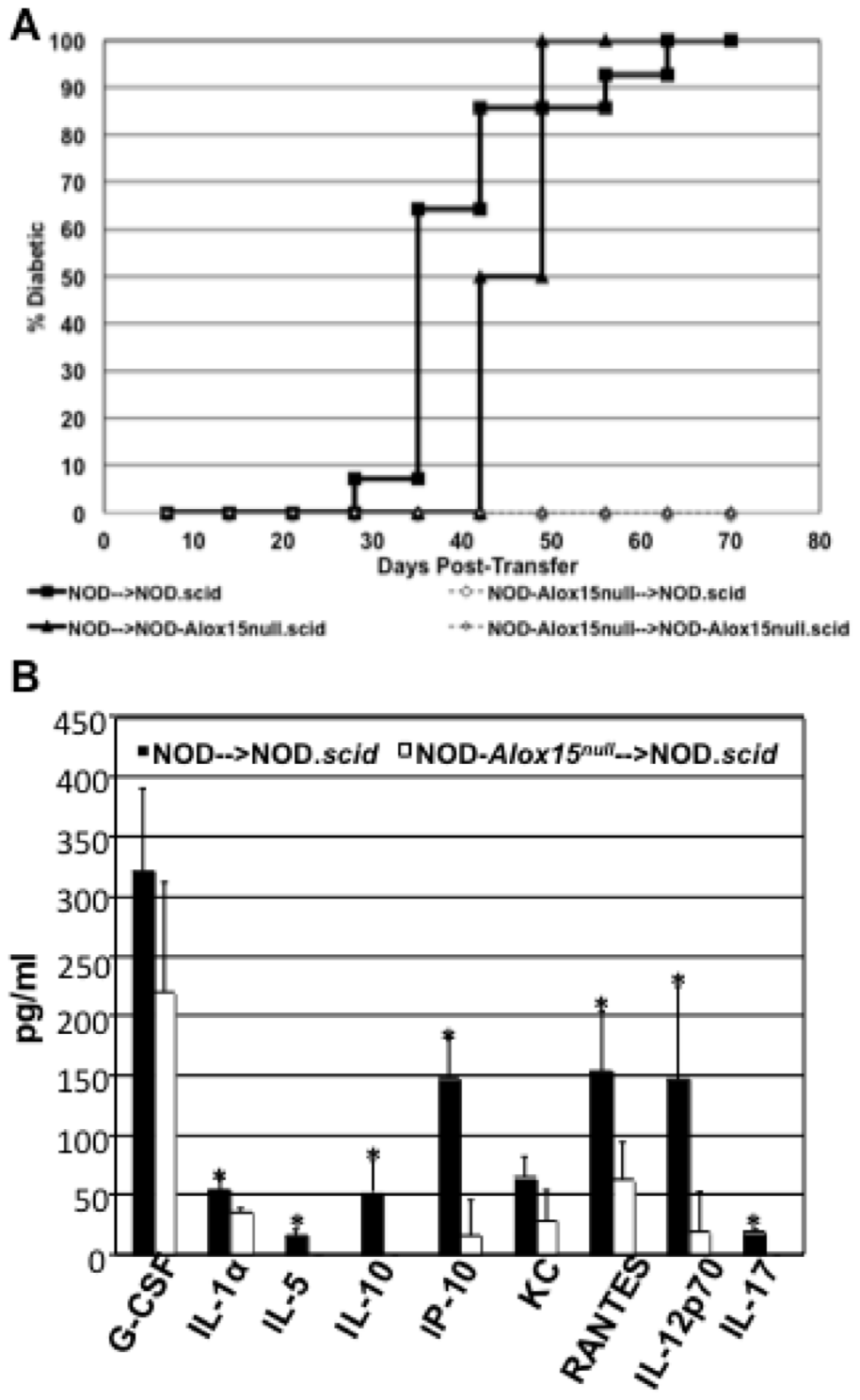
Adoptive transfer of NOD and NOD-*Alox15^null^* splenocytes. Donor cells were either non-diabetic NOD or NOD-*Alox15^null^* donor splenocytes transferred into NOD.*scid* or diabetic NOD and non-diabetic NOD-*Alox15^null^* donor splenocytes transferred into NOD-*Alox15^null^.scid* recipients. All donors were at least 13 weeks old at the time of transfer. C) Total serum cytokine measurements of adoptive transfer hosts. Data are pooled from either 2 (NOD-*Alox15^null^.scid* recipients) or 3 (NOD.*scid* recipients) experiments. For NOD versus NOD-*Alox15^null^* donor splenocytes into NOD.*scid* mice, p≤0.0001, or p≤0.0003 for NOD-*Alox15^null^*.*scid* recipients. NOD into NOD.*scid* recipients. Difference between recipients given NOD donor cells, p≤0.03.

To test whether deletion of 12/15 LO in macrophages affects T cell ability to transfer disease, we performed adoptive transfer assays by injecting unfractionated splenocytes from either NOD or NOD-*Alox15^null^* mice into NOD.*scid* or NOD-*Alox15^null^.scid* mice (Figure 2A). These results show that pre-diabetic NOD donor splenocytes are able to transfer disease to both NOD.*scid* and NOD-*Alox15^null^.scid* recipients (Figure 2A). In contrast, splenocytes from NOD-*Alox15^null^* donors did not transfer disease. Interestingly, NOD-*Alox15^null^.scid* recipients showed a slight, but statistically significant delay in disease onset compared to NOD.*scid* recipients following the adoptive transfer of NOD splenocytes (p<0.03 by the Wilcoxon test). Consistent with a dramatic difference in disease transmission, serum cytokines from recipients given NOD donor cells showed significant increases in IL-1α, IL-5, IL-10, IP-10, RANTES, IL-12p70, and IL-17A compared to those given NOD-*Alox15^null^* donor cells (Figure 2C).

**Figure 3:**
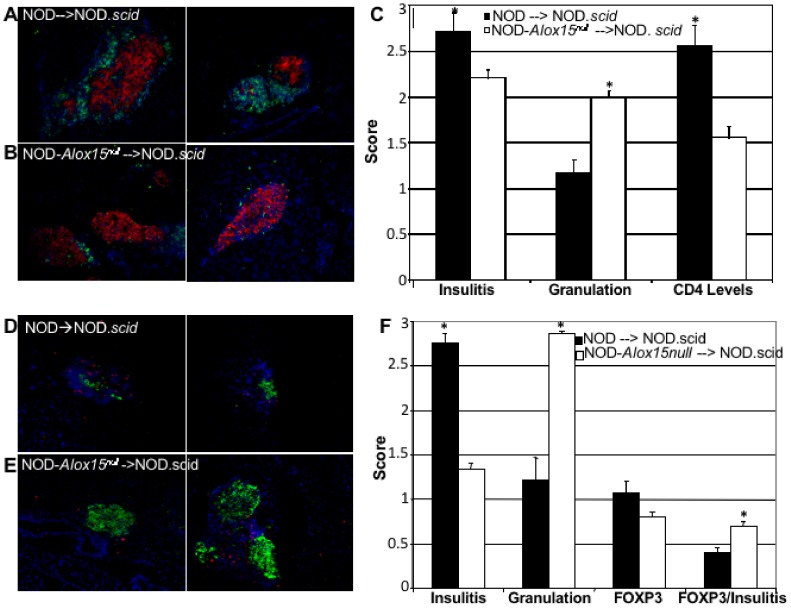
CD4^+^ T cell infiltration after adoptive transfer. Representative pictures of frozen pancreas sections stained with anti-CD4 (green), anti-insulin (red), and Hoechst nuclear dye (blue) from NOD.*scid* host mice receiving either (A) NOD (n = 5) or (B) NOD-*Alox15^null^* (n = 5) donor cells. Results are quantified in (C). Foxp3^+^ T cells are present after adoptive transfer. Representative pictures of frozen pancreas sections stained with anti-Foxp3 (red), anti-insulin (green), and Hoechst nuclear dye (blue) from NOD.*scid* host mice receiving either (D) NOD (n = 5) or (E) NOD-*Alox15^null^* (n = 5) donor splenocytes. (F) Quantified results with the presence of Foxp3 expressed in a ratio with insulitis levels. *p<0.05. These data represent 3 experiments with n = 5 mice per group.

Further characterization of the infiltration in adoptive transfer hosts allowed us to determine that CD4^+^ T cells are significantly increased in NOD.*scid* mice injected with NOD donor cells (Figure 3A) as compared to those injected with NOD-*Alox15^null^* donor splenocytes (Figure 3B). Frozen pancreas sections were stained with anti-insulin (red) and anti-CD4 antibodies (green), as well as with the Hoechst nuclear stain (blue). Representative sections are shown in Figure 3A and B. These studies showed that insulitis, or the levels of infiltrating mononuclear cells, was significantly increased, and comprised of CD4^+^ T cell infiltration. Granulation, which is an indicator of the amount of insulin stored in beta cell granules, was significantly lower in NOD.*scid* hosts receiving NOD splenocytes (quantified in Figure 3C). Sections were also stained with anti-insulin (green) and anti-Foxp3 antibodies (red) and Hoechst nuclear stain (blue) in order to assess the levels of regulatory T cells following adoptive transfer of either NOD or NOD-*Alox15^null^* donor splenocytes (Figure 3D & E). When Foxp3 was globally scored, we saw no difference between the two groups. However, we investigated the presence of Foxp3^+^ cells in the context of insulitis and found that there was a significant increase in Foxp3^+^ cells when additional insulitis was present (Figure 3F). These data demonstrate that, in the presence of infiltrating lymphocytes, there is a significant increase in the levels of Foxp3^+^ cells. This may indicate that 12/15-LO negatively affects the development or recruitment of regulatory T cells during the islet inflammatory processes of type 1 diabetes.

### 12/15-LO Expression Affects Islet Mass and Functional Gene Expression

**Figure 4:**
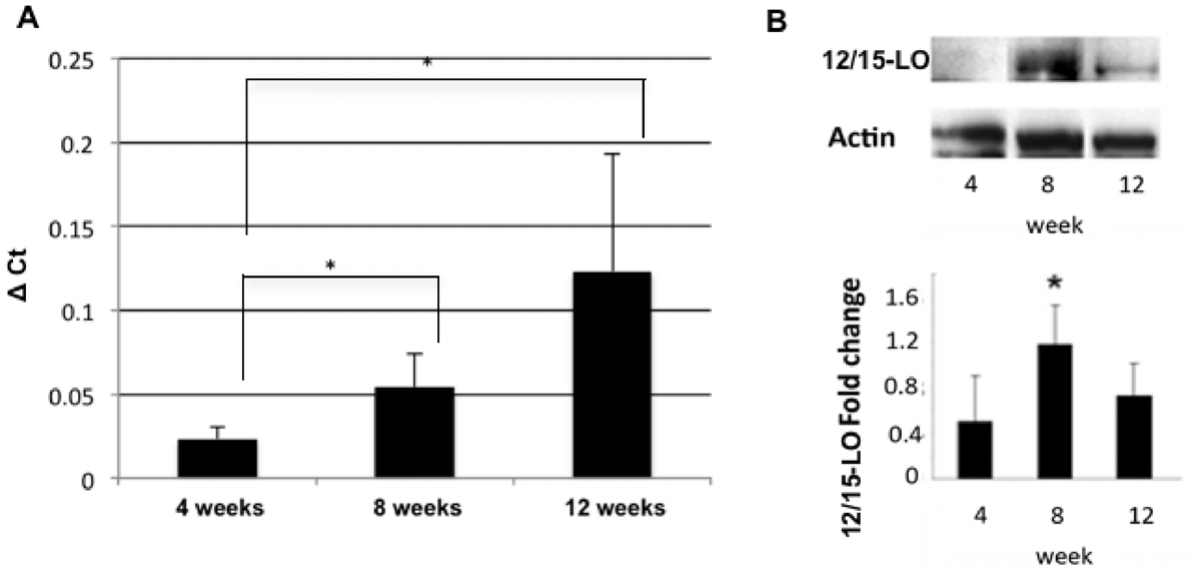
12/15-Lipoxgenase levels in pancreatic islets of NOD mice. Pancreatic islets were isolated from NOD mice at 4, 8, and 12 weeks of age. 12/15-LO expression was measured both by (A) qRT-PCR or (B) Western blotting. *, p<0.05. All samples are normalized to actin control. These data represent 3 experiments with n = 6 for each strain of mice at each time point.

We next wanted to explore 12/15-LO expression in islets. Previous studies have shown 12/15-LO is expressed in both mouse and human islets [8], [14], [15]. NOD islets show 12/15-LO expression at both the mRNA and protein level (Figure 4). mRNA expression increased steadily and significantly as the mice aged (Figure 4A), while protein levels peaked at 8 weeks of age (Figure 4B).

**Figure 5:**
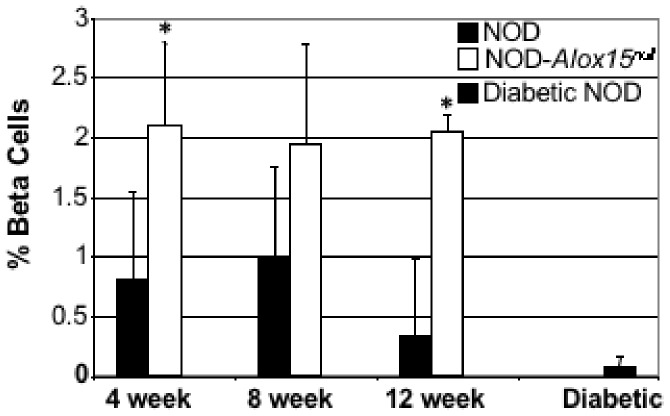
Beta-cell area of NOD versus NOD-*Alox15^null^* pancreata. *p<0.05 age-matched NOD vs. NOD-*Alox15^null^* ^.^ Filled bars represent NOD mice and open bars represent NOD-*Alox15^null^* mice. Data represent the average from 3 sections per pancreas, and 3–5 pancreata from each group.

Investigations into islet viability suggested that NOD-*Alox15^null^* mice have more viable islets for a longer period of time. Calculation of the percent of beta cells contributing to the total pancreatic area shows that absence of 12/15-LO is associated with increased beta cell mass in NOD-*Alox15^null^* mice (Figure 5). Even at 4 weeks of age, NOD-*Alox15^null^* mice exhibit a significantly higher percentage of beta cells per total pancreatic area as compared to NOD mice. The percentage of beta cells per total pancreas section remained similar as the NOD-*Alox15^null^* mice aged, but decreased in NOD mice as they aged and became diabetic.

Focused gene expression was used to ascertain islet health on a molecular level. Islets from the two NOD background strains (n = 4 per strain per time point) were tested by qRT-PCR for mRNA expression of the islet-specific factors (GLUT2, Pdx1), and the mitochondrial stress factor UCP2 (Table 1). GLUT2 mRNA expression was similar in both NOD and NOD-*Alox15^null^* islets at 4 and 8 weeks. However, at 12 weeks, the expression of GLUT2 in NOD-*Alox15^null^* islets was significantly higher than in wild type NOD islets. Pdx1 mRNA expression was readily detected in islets from both strains at 4 weeks, but in NOD islets, Pdx1 expression decreased significantly by 8 weeks. However, Pdx1 expression increased steadily in NOD-*Alox15^null^* islets as the mice aged. Gene expression of the mitochondrial stress factor UCP2 increased slightly as wild type NOD mice aged, but remained relatively level in NOD-*Alox15^null^* islets.

**Table 1:** mRNA expression of molecular indicators of islet health in NOD and NOD-*Alox15^null^* mouse islets.

Gene	4 weeks		8 weeks		12 weeks	
	NOD-*Alox15^null^*	NOD	NOD-*Alox15^null^*	NOD	NOD-*Alox15^null^*	NOD
GLUT2	1.0±0.6^b^	1.2±0.4^b^	0.5±0.03^b^	0.5±0.5^b^	2.8±0.5^a^	0.3±0.1
Pdx1	1.0±0.5^b^	1.0±0.2^b, c^	2.2±0.2^a, b^	0.2±0.07	4.3±0.6^a^	0.2±0.04
UCP2	1.0±0.2^c^	1.7±1.2	1.3±0.06	1.0±0l7^b^	1.2±0.6	4.0±1.6

Data are shown as fold change based on control (4 week old NOD-*Alox15^null^* samples) and normalized to Actin. All samples were run in triplicate with an (n = 4). ^a^p<0.05 vs age-matched groups (i.e., 8 wk NOD vs. 8 wk NOD-*Alox15^null^*); ^b^p<0.05 vs. 12 week time point in strain-matched groups (i.e., 8 wk NOD vs. 12 wk NOD); ^c^p<0.05 vs. 8 week time point in strain-matched groups (i.e., 4 wk NOD vs. 8 wk NOD).

**Figure 6:**
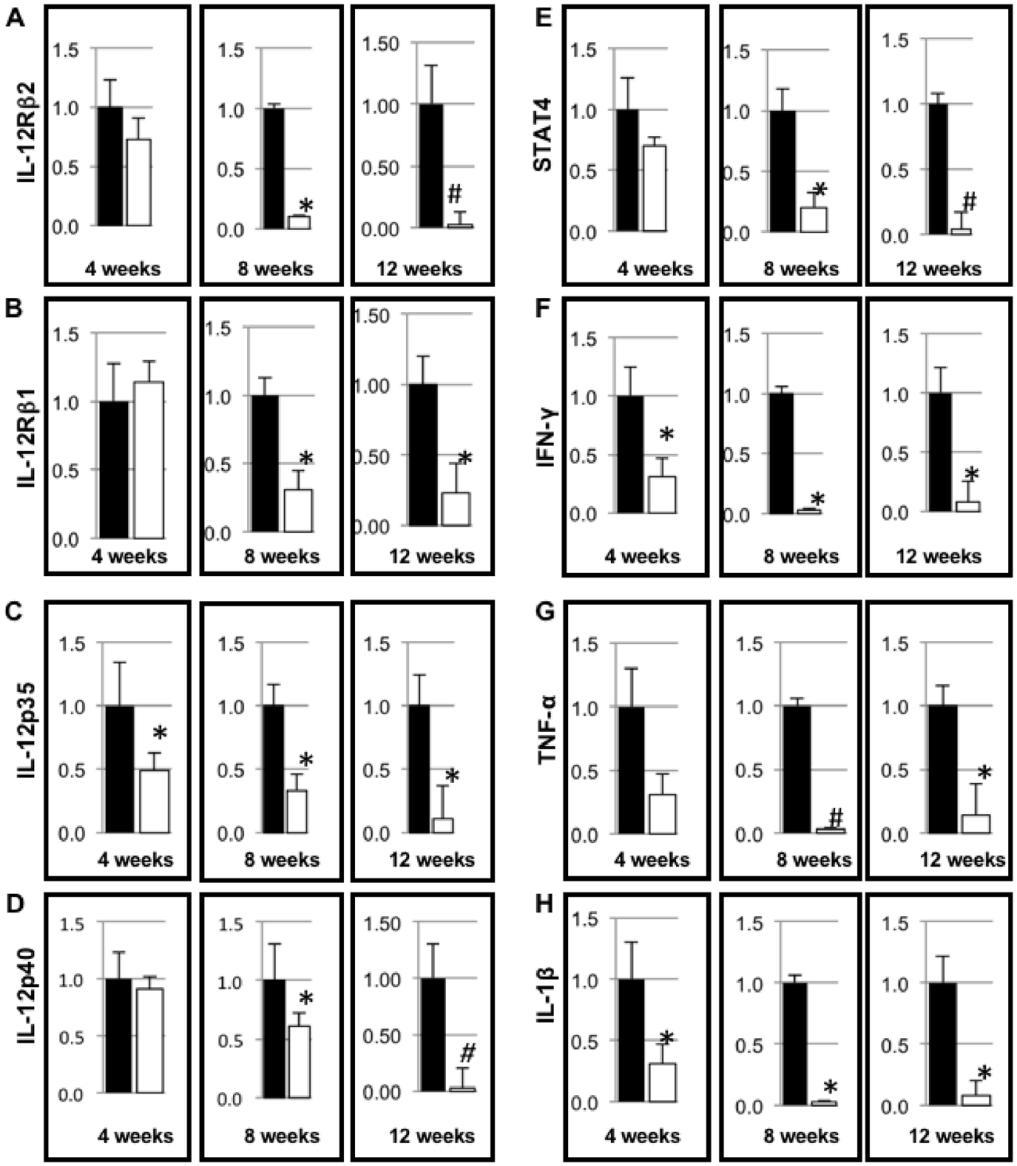
Gene expression of pro-inflammatory cytokines related to the IL-12/12/15LO axis. A) mRNA expression of IL-12Rβ2, B) IL-12Rβ1, C) IL-12p35, D) IL-12p40, E) STAT4, F) IFN-γ, G) TNF-α, and H) IL-1β in 4, 8, and 12 week old NOD and NOD-*Alox15^null^* mice islets. All samples were run in triplicate with an (n = 6). Data are expressed as fold reduction compared to their aged matched control (NOD). *p<0.05 and ^#^p<0.005 for NOD vs. NOD-*Alox15^null^* at the same age. Filled bars represent NOD and open bars represent NOD-*Alox15^null^* samples.

A TH17 PCR array was used to evaluate key inflammatory cytokines in islets from *Alox15^null^* and wild type female NOD mice (Table S1, n = 3 mice per strain per time point). The mRNA levels of several molecules were decreased in the absence of 12/15-LO in islets. Validation of the TH17 array by qRT-PCR demonstrated two different expression patterns for the genes tested. In NOD-*Alox15^null^* islets, IL-12Rβ2, IL-12p35, IL-12p40, and STAT4 all decreased over time, with significant decreases seen by 8 weeks compared to NOD islets (Figure 6A, C, D, and E). In the second pattern grouping, expression levels reached a nadir at 8 weeks, followed by a slight increase at 12 weeks, with levels still remaining lower than age-matched NOD controls. IL-12Rβ1, IFN-γ, TNF-α, and IL-1β fall into this group (Figures 6B, F, G, and H). These results show that islet-specific proinflammatory cytokine responses are significantly blunted in the absence of 12/15-LO in NOD-*Alox15^null^* mice compared to NOD controls, indicating a clear role for the 12/15 lipoxygenase gene in the development of inflammatory responses in T1D.

**Figure 7:**
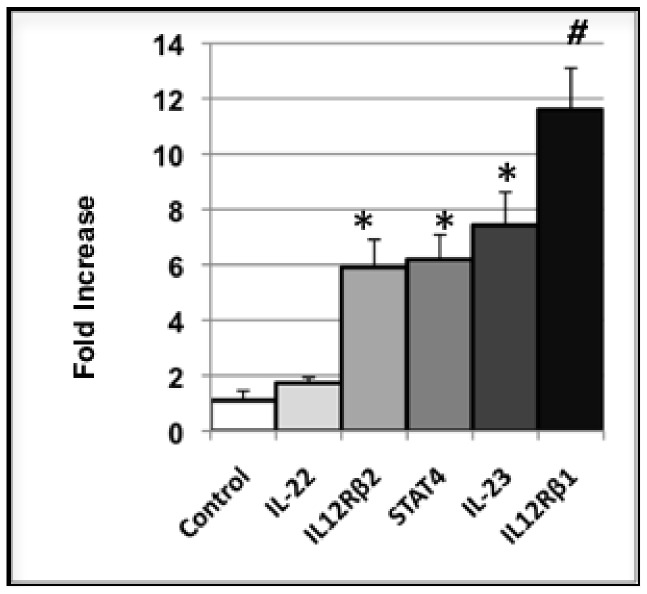
Cytokine production in triple cytokine-treated βTC3 cells. βTC3 cells were treated with a triple cytokine cocktail (IFN-γ, TNF-α, and IL-1β). Cytokine production was measured, and data are expressed as the fold change over paired untreated controls, with n = 8 for each sample. These data represent 3 separate experiments.

While some of the increased inflammation in NOD islets is due to the presence of immune cells (i.e.,10-fold increase in CD3e mRNA over NOD-*Alox15^null^* islets), a portion of the inflammatory response starts in the islet itself. Since beta cells cannot be separated from infiltrating cells in NOD mouse islets, we utilized a murine beta cell line, βTC3, to test for beta cell-specific cytokine expression under normal and inflammatory conditions. βTC3 cells show an increase in IL-23p19, IL-12Rβ1 & 2, and STAT4 following triple cytokine treatment (IFN-γ, TNF-α, and IL-1β) (Figure 7). IL-22, which is produced only by lymphoid cells [26], does not change following the same treatment. Data are expressed as fold change of each target over untreated, paired controls (n = 8).

We found no significant differences in 12/15-LO or cytokine mRNA expression in the spleen, lymph nodes, or fat for any of the genes tested in NOD and NOD-*Alox15^null^* tissues at 4, 8, and 12 weeks of age (Figure S1, data not shown).

### Functional Assays Reveal that IL-12 Negatively Affects Islet Health

**Figure 8:**
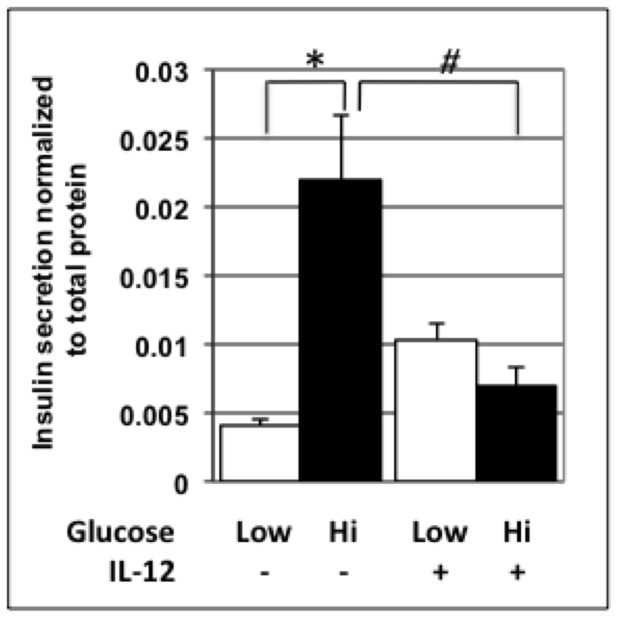
GSIS of control versus IL-12p70-treated MIN6 cells. MIN6 cells were incubated in the presence or absence of IL-12 under high and low glucose conditions, and GSIS was measured following this incubation. Open bars are incubated under low glucose conditions, while filled bars are incubated with high glucose conditions. N = 5 samples/treatment. Data represent 3 separate experiments.

IL-12p70 is a downstream product of the 12/15-LO pathway [6], and is decreased in the absence of 12/15-LO. Since we demonstrated that mRNA levels of both IL-12 subunits and their respective receptors were increased in NOD over NOD-*Alox15^null^* islets, we wanted to isolate potential effects on isolated beta cells. Therefore, we tested the effect of IL-12 on the MIN6 beta cell line, which exhibits characteristics of GSIS similar to those of normal islets [25]. MIN6 cells treated with 50 ng/ml of IL-12 have impaired insulin secretion in response to both low and high levels of glucose (Figure 8). Data are expressed as insulin secretion as normalized to total cellular protein levels. Additional evidence from our group suggests that this is also true in rat and human beta cells [27].

## Discussion

The goal of our studies has been to identify which source of 12/15-LO is more important in the pathogenesis of T1D. The key finding of the present study is that global deletion of *Alox15* modified both immune cell and islet components in NOD mice. NOD-*Alox15^null^* mice exhibit significant reduction in pancreatic inflammation as compared to age-matched NOD mice. Subsequently, this translated to preserved beta cell volume in NOD-*Alox15^null^* mice.

We found that macrophages, but not B and T cells, express 12/15-LO mRNA. This is consistent with previous studies in other models showing expression of 12/15LO in macrophages, but not in Tor B cells [9], [13]. Together, with our previous study of 12/15-LO mRNA and protein levels in NOD macrophages, it was not surprising to find that 12/15-LO deficient macrophages produce significantly reduced amounts of STAT4 and IL-12p40, which are downstream of 12/15-LO. Surprisingly, MCP-1 levels did not vary between the two NOD strains. Given previous results, this was unexpected [28]. It is possible that strain differences between NOD and C57BL/6 mice lead to this difference.

Functionally, it appears that *Alox15^null^* macrophages are unable to stimulate the down stream autoimmune response as tested by adoptive transfer. 12/15-LO expression levels, presumably in macrophages or dendritic cells in the NOD mouse model, likely affect the ability of T cells to transfer disease. NOD-derived donor splenocytes are capable of transferring T1D, even when originating from a non-diabetic mouse. In contrast, non-diabetic NOD-*Alox15^null^* donor cells are unable to transfer disease, suggesting that macrophage *Alox15* expression greatly influences the development of the autoimmune response in the NOD mouse strain. This influence may be exerted through the expression and signaling of IL-12 and STAT4, which are key signals driving Th1 cell development. In the absence of 12/15-LO, IL-12 production and STAT4 signaling are significantly reduced. The absence of STAT4, in both streptozotocin-induced and spontaneous diabetes, protects against the development of diabetes [11], [29], [30]. Additionally, lisofylline, which inhibits STAT4 activation, also provides protection against diabetes development [31]. Reductions in IL-12 and STAT4 signaling have been shown to reduce the induction of T-bet, and subsequently production of IFN-γ [32], [33]. This type of reduction could result in the skewing towards more protective Th2-like immune responses. Our data indicate that these effects include alterations in Foxp3^+^ regulatory T cells, which are increased around the islets during insulitis in the absence of 12/15-LO [4]. It is possible that the increase in Foxp3^+^ cells aids in the prevention of diabetes progression in recipients receiving NOD-*Alox15^null^* donor cells. The rate of T1D development after adoptive transfer of splenocytes from NOD mice slowed slightly, but significantly, in NOD-*Alox15^null^.scid* mice, indicating that a lack of 12/15-LO expression in islets may slow disease progression; however, in the presence of fully activated autoimmune systems, and activated Th1 cells, this lack of expression does not afford full protection to the islets. Therefore, it appears that macrophage 12/15-LO expression plays an important role in initiating the autoimmune response.

Although 12/15-LO plays a vital role in directing macrophage function, we have also shown that 12/15-LO levels are increased in NOD islets as early as 8 weeks of age. This is consistent with the typical age of onset of most inflammatory processes in the pathogenesis of T1D of NOD mice. Indeed, this is the time during which circulating auto-reactive cells undergo significant expansion [34], and decreased beta cell function becomes more evident [35]. Since 12/15-LO was found in murine and human islets [14], [15], we felt it was pertinent to address subsequent changes in islets as a result of this expression. To investigate cellular stress of islets, we measured gene expression of GLUT2, Pdx1, and UCP2 [16]. GLUT2, important in glucose sensing by beta cells, was increased in NOD-*Alox15^null^* islets compared to age-matched wild-type NOD mice, which complements the increased beta cell area seen in NOD-*Alox15^null^* mice. NOD-*Alox15^null^* mouse islets exhibited a steady increase in Pdx1, a transcription factor responsible for beta cell maturation and pancreatic development [36], as the mice aged. NOD mouse islets, however, showed a down-regulation in the Pdx1 gene as the mice progressed toward overt diabetes. These changes may be explained in part by the decrease in beta cell mass caused by autoimmune insulitis. However, it is unclear why Pdx1 mRNA levels drop off more rapidly than beta cell mass and insulin levels at 8 weeks of age. Perhaps the Pdx1 protein is more stable than the message at that point in time. Recent studies have shown that Pdx1 protein is decreased at 10 weeks of age in NOD islets [16], but the levels at 8 weeks were not studied. Further studies are needed to confirm this hypothesis. UCP2, an uncoupling protein in the inner mitochondrial membrane, is responsible for the production of ATP during oxidative phosphorylation in the electron transport chain, and is essential for insulin secretion [37]. The overexpression of UCP2 at 12 weeks in the NOD mice may reflect overcompensation of UCP2 in an attempt to override the impaired glucose secretion, or it may be a side effect of cellular stress caused by the highly inflamed environment.

Proinflammatory cytokines increased in the islets of aging NOD mice, but not in NOD-*Alox15^null^* mice. Mediators from the IL-12/IFN-γ pathway (IFN-γ, IL-12p40, IL-12p35, IL-12Rβ1, IL-12Rβ2 and STAT4), TNF-α, and IL-1β were all significantly down-regulated in NOD-*Alox15^null^* islets**.** In addition, NOD-*Alox15^null^* mouse islets did not show an accumulation of Th1/Th17 cytokines as they aged compared to the NOD mouse islets. Many of these cytokines are involved in mediating inflammatory responses. Up-regulation of these cytokines in the 12 week-old NOD mouse islets is consistent with the idea that IL-12 axis cytokines are major contributors to the destruction of beta cells in T1D [38]. The exact roles of these cytokines in T1D will require further study, but it is of interest that deletion of 12/15-LO reduced cytokine expression in NOD-*Alox15^null^* islets. Although some of these cytokines may be attributed to the lymphocytic infiltration in the islets at the time of harvest, we have also shown that mouse beta cells themselves are capable of producing inflammatory cytokines, as βTC3 cells treated with a triple cytokine cocktail show increased cytokine and cytokine receptor expression following treatment.

Functional analysis of the effects of IL-12, a downstream product of 12/15-LO activity, showed that IL-12 impairs GSIS at both low and high levels of glucose in the MIN6 beta cell line. These studies point to downstream effects of 12/15-LO expression in the pancreas. Recent data from our group show a direct role for IL-12 and its receptors in the development of islet inflammation. These studies were confirmed in several beta cell lines [27].

Our work suggests that macrophage and islet 12/15-LO both play a role in the development of the inflammatory processes in the pancreas. Inflammation appears to be required for the initiation of diabetes in NOD mice [39], and 12/15-LO may play a vital role in the development of this inflammation. Indeed, a recent study has shown that inhibition of 12-lipoxygenase decreases NADPH-oxidase 1, as well as endogenous reactive oxygen species, which protects beta cell lines and cultured islets from apoptosis [40]. As 12-lipoxygenase is upstream of these mediators, it is a strong therapeutic candidate. Additional studies are needed to define the particular role of 12/15-LO in the islet as compared to the macrophage. Macrophage-specific and islet-specific deletions of *Alox15* in NOD will be helpful in clarifying this issue.

Overall, our data point to a model in which 12/15-LO affects islet health early in development. Since 12/15-LO is involved in signaling leading to ER stress, it is possible that early unknown events trigger the inflammatory signaling cascade [41], which could cause the recruitment and activation of macrophages. As macrophages are also a source of 12/15-LO, their production could exacerbate the cycle, leading to additional inflammation. Since human islets express 12-LO, it is possible that targeting this enzyme may provide a new therapeutic option to help maintain beta cell mass in T1D.

## Supporting Information

Figure S1 **12/15-LO mRNA expression in NOD tissues (Lymph nodes (A), fat and spleen (B)). N = 6 per time point.** Data are representative of 3 experiments.
(TIFF)

Table S1 **Th17 array of NOD and NOD-** ***Alox15^null^*** **islets at 8, 12, and 16 weeks.** N = 3 per group, per time point.
(DOCX)
