# Supplementary material for: Deletion of 12/15-Lipoxygenase Alters Macrophage and Islet Function in NOD-Alox15null Mice, Leading to Protection against Type 1 Diabetes Development
Source: PLoS One. 2013 Feb 21;8(2):e56763. doi: 10.1371/journal.pone.0056763 (PMC3578926; doi:10.1371/journal.pone.0056763)
Supplement: Table S1 — Th17 array of NOD and NOD- Alox15null islets at 8, 12, and 16 weeks. N = 3 per group, per time point. (DOCX) [file pone.0056763.s002.docx]

| TABLE S1. |  |  | **Fold change (n=3)** | **Fold change (n=3)** | **Fold change (n=3)** |
| --- | --- | --- | --- | --- | --- |
| **Unigene** | **Refseq** | **Gene** | **8wk NOD VS 8wkNULL** | **12wk NOD VS 12wkNULL** | **16wk NOD VS 16wkNULL** |
| Mm.10702 | NM_009786 | Cacybp | 0.7213 | 0.8645 | 3.1467 |
| Mm.1283 | NM_011329 | Ccl1 | 0.8019 | 0.4714 | 5.0183 |
| Mm.290320 | NM_011333 | Ccl2 | 1.4203 | 0.8526 | 0.9332 |
| Mm.116739 | NM_016960 | Ccl20 | 0.8019 | 0.5105 | 1.6158 |
| Mm.12895 | NM_009137 | Ccl22 | 0.8626 | 1.3755 | 1.9691 |
| Mm.341574 | NM_013654 | Ccl7 | 1.2463 | 1.2184 | 1.4958 |
| Mm.22842 | NM_013486 | Cd2 | 0.5924 | 1.7839 | 4.6289 |
| Mm.217308 | NM_031162 | Cd247 | 1.0193 | 0.7195 | 1.9484 |
| Mm.255003 | NM_007642 | Cd28 | 1.5057 | 10.8905 | 7.8824 |
| Mm.29798 | NM_133654 | Cd34 | 0.3976 | 1.3519 | 2.9067 |
| Mm.4527 | NM_013487 | Cd3d | 1.4574 | 20.2521 | 7.3258 |
| Mm.210361 | NM_007648 | Cd3e | 0.8852 | 10.0561 | 11.3181 |
| Mm.335106 | NM_009850 | Cd3g | 1.0032 | 6.3203 | 13.0728 |
| Mm.2209 | NM_013488 | Cd4 | 1.3777 | 10.3031 | 8.7905 |
| Mm.4861 | NM_011616 | Cd40lg | 0.5777 | 0.2698 | 6.1754 |
| Mm.1858 | NM_001081110 | Cd8a | 1.3173 | 8.9693 | 10.8489 |
| Mm.439656 | NM_009883 | Cebpb | 1.1206 | 0.4948 | 1.082 |
| Mm.239516 | NM_020008 | Clec7a | 1.7891 | 5.3332 | 2.6935 |
| Mm.4922 | NM_009969 | Csf2 | 2.8326 | 0.9298 | 0.2971 |
| Mm.1238 | NM_009971 | Csf3 | 0.4964 | 1.1728 | 0.7752 |
| Mm.103711 | NM_009142 | Cx3cl1 | 0.7538 | 0.4506 | 0.7922 |
| Mm.21013 | NM_008176 | Cxcl1 | 1.5476 | 0.7245 | 0.3483 |
| Mm.303231 | NM_021704 | Cxcl12 | 0.7469 | 2.2038 | 4.0441 |
| Mm.4979 | NM_009140 | Cxcl2 | 1.5674 | 0.6462 | 0.3596 |
| Mm.4660 | NM_009141 | Cxcl5 | 4.6735 | 0.732 | 0.2031 |
| Mm.90154 | NM_080729 | Il25 | 0.8019 | 0.2902 | 1.1113 |
| Mm.982 | NM_007901 | S1pr1 | 0.8274 | 1.257 | 1.8668 |
| Mm.182291 | NM_054039 | Foxp3 | 0.8019 | 0.4019 | 3.7005 |
| Mm.313866 | NM_008091 | Gata3 | 0.8696 | 4.1554 | 3.9149 |
| Mm.435508 | NM_010493 | Icam1 | 0.5207 | 0.933 | 1.477 |
| Mm.42044 | NM_017480 | Icos | 1.8672 | 23.1029 | 4.1502 |
| Mm.240327 | NM_008337 | Ifng | 2.5982 | 5.0281 | 20.1103 |
| Mm.874 | NM_010548 | Il10 | 0.9467 | 1.834 | 4.1227 |
| Mm.239707 | NM_008352 | Il12b | 0.4859 | 5.5713 | 6.904 |
| Mm.731 | NM_008353 | Il12rb1 | 1.0611 | 7.4191 | 4.2117 |
| Mm.188337 | NM_008354 | Il12rb2 | 3.1814 | 15.6108 | 3.2707 |
| Mm.1284 | NM_008355 | Il13 | 0.7332 | 0.3499 | 1.1822 |
| Mm.4392 | NM_008357 | Il15 | 1.6191 | 1.7901 | 2.2481 |
| Mm.5419 | NM_010552 | Il17a | 0.8019 | 0.2822 | 0.9599 |
| Mm.222808 | NM_145834 | Il17c | 0.7357 | 0.3356 | 2.6813 |
| Mm.390726 | NM_145837 | Il17d | 1.1353 | 0.7846 | 2.6396 |
| Mm.222807 | NM_145856 | Il17f | 0.59 | 0.5724 | 2.0173 |
| Mm.269363 | NM_019583 | Il17rb | 0.5732 | 0.5548 | 2.1747 |
| Mm.213397 | NM_134159 | Il17rc | 0.6285 | 0.5967 | 2.0413 |
| Mm.206726 | NM_134437 | Il17rd | 0.7889 | 0.3427 | 1.5506 |
| Mm.131781 | NM_145826 | Il17re | 0.4096 | 0.8919 | 2.6174 |
| Mm.1410 | NM_008360 | Il18 | 1.0028 | 1.0534 | 3.283 |
| Mm.222830 | NM_008361 | Il1b | 1.1954 | 5.2964 | 7.1641 |
| Mm.14190 | NM_008366 | Il2 | 6.0102 | 9.1814 | 1.8243 |
| Mm.157689 | NM_021782 | Il21 | 0.8019 | 0.5141 | 1.5392 |
| Mm.103585 | NM_016971 | Il22 | 2.72 | 131.5918 | 8.6434 |
| Mm.125482 | NM_031252 | Il23a | 0.3387 | 1.2483 | 0.3958 |
| Mm.221227 | NM_144548 | Il23r | 0.9983 | 1.8025 | 2.4868 |
| Mm.222632 | NM_145636 | Il27 | 0.8462 | 3.1602 | 10.3364 |
| Mm.983 | NM_010556 | Il3 | 0.8019 | 0.2902 | 1.1113 |
| Mm.276360 | NM_021283 | Il4 | 0.8019 | 0.2902 | 1.1113 |
| Mm.4461 | NM_010558 | Il5 | 1.4994 | 1.4389 | 0.9914 |
| Mm.1019 | NM_031168 | Il6 | 1.0963 | 0.1066 | 1.3084 |
| Mm.2856 | NM_010559 | Il6ra | 0.7156 | 1.3426 | 2.9427 |
| Mm.389 | NM_008372 | Il7r | 2.0481 | 0.6263 | 2.0709 |
| Mm.322843 | NM_020583 | Isg20 | 1.2359 | 29.446 | 2.327 |
| Mm.289657 | NM_146145 | Jak1 | 0.9735 | 1.0497 | 2.1638 |
| Mm.275839 | NM_008413 | Jak2 | 1.0528 | 1.7112 | 3.2492 |
| Mm.5022 | NM_008607 | Mmp13 | 2.1863 | 1.057 | 1.0085 |
| Mm.4993 | NM_010809 | Mmp3 | 4.4101 | 0.4763 | 0.1163 |
| Mm.4406 | NM_013599 | Mmp9 | 0.8019 | 0.2902 | 0.5355 |
| Mm.213003 | NM_010851 | Myd88 | 0.8525 | 1.1329 | 4.1992 |
| Mm.116802 | NM_010899 | Nfatc2 | 0.5725 | 1.5476 | 5.396 |
| Mm.256765 | NM_008689 | Nfkb1 | 1.0369 | 1.4191 | 2.1955 |
| Mm.4372 | NM_011281 | Rorc | 0.8953 | 0.9044 | 4.373 |
| Mm.130 | NM_009896 | Socs1 | 0.9377 | 3.2266 | 7.8358 |
| Mm.3468 | NM_007707 | Socs3 | 0.8805 | 1.429 | 3.3078 |
| Mm.249934 | NM_011486 | Stat3 | 0.8031 | 1.257 | 3.1131 |
| Mm.1550 | NM_011487 | Stat4 | 2.9319 | 6.0202 | 1758.3497 |
| Mm.277403 | NM_011488 | Stat5a | 1.2603 | 1.8277 | 2.0106 |
| Mm.121721 | NM_009284 | Stat6 | 0.8227 | 0.0369 | 1.5935 |
| Mm.375031 | NM_011518 | Syk | 1.1294 | 3.1602 | 4.4783 |
| Mm.477879 | NM_019507 | Tbx21 | 0.8019 | 0.8039 | 3.2682 |
| Mm.248380 | NM_011577 | Tgfb1 | 0.7743 | 1.2311 | 2.9304 |
| Mm.23987 | NM_054096 | Tirap | 0.7587 | 0.5212 | 1.3182 |
| Mm.38049 | NM_021297 | Tlr4 | 0.4066 | 1.1933 | 2.3702 |
| Mm.1293 | NM_013693 | Tnf | 0.3622 | 1.8232 | 6.3065 |
| Mm.292729 | NM_009424 | Traf6 | 0.8095 | 0.8827 | 1.8965 |
| Mm.3868 | NM_009537 | Yy1 | 0.6541 | 0.8039 | 1.4213 |
| Mm.3317 | NM_010368 | Gusb | 0.6811 | 0.7658 | 2.576 |
| Mm.299381 | NM_013556 | Hprt1 | 0.8906 | 0.917 | 2.102 |
| Mm.2180 | NM_008302 | Hsp90ab1 | 1.1278 | 0.446 | 0.6904 |
| Mm.343110 | NM_008084 | Gapdh | 1 | 1 | 1 |
| Mm.328431 | NM_007393 | Actb | 0.7053 | 1.0425 | 2.6754 |
| N/A | SA_00106 | MGDC | 0.8019 | 0.2902 | 1.1113 |
| N/A | SA_00104 | RTC | 0.8019 | 0.2902 | 1.1151 |
| N/A | SA_00104 | RTC | 0.8019 | 0.2902 | 1.1113 |
| N/A | SA_00104 | RTC | 0.8019 | 0.2902 | 1.1113 |
| N/A | SA_00103 | PPC | 0.7549 | 0.2615 | 1.211 |
| N/A | SA_00103 | PPC | 0.7261 | 0.257 | 1.1983 |
| N/A | SA_00103 | PPC | 0.7868 | 0.2774 | 1.2679 |
|  |  |  |  |  |  |

Table S1: Th17 array of NOD and NOD-*Alox15^null^* islets at 8, 12, and 16 weeks. N=3 per group, per time point.
